# Testing a deliberative democracy method with citizens of African ancestry to weigh pros and cons of targeted screening for hereditary breast and ovarian cancer risk

**DOI:** 10.3389/fpubh.2022.984926

**Published:** 2022-11-08

**Authors:** Yue Guan, Sarita Pathak, Denise Ballard, J. K. Veluswamy, Lauren E. McCullough, Colleen M. McBride, Michele C. Gornick

**Affiliations:** ^1^Department of Behavioral, Social, and Health Education Sciences, Rollins School of Public Health, Emory University, Atlanta, GA, United States; ^2^Winship Cancer Institute of Emory University, Atlanta, GA, United States; ^3^Horizons Community Solutions, Albany, GA, United States; ^4^Department of Epidemiology, Rollins School of Public Health, Emory University, Atlanta, GA, United States

**Keywords:** public engagement, health policy, stakeholder participation, democratic deliberation, hereditary cancer syndromes, minority groups

## Abstract

**Background:**

Democratic deliberation (DD), a strategy to foster co-learning among researchers and communities, could be applied to gain informed public input on health policies relating to genomic translation.

**Purpose:**

We evaluated the quality of DD for gaining informed community perspectives regarding targeting communities of African Ancestry (AAn) for Hereditary Breast and Ovarian Cancer (HBOC) screening in Georgia.

**Methods:**

We audiotaped a 2.5 day conference conducted via zoom in March 2021 to examine indicators of deliberation quality based on three principles: (1) inclusivity (diverse viewpoints based on participants' demographics, cancer history, and civic engagement), (2) consideration of factual information (balanced and unbiased expert testimonies, participant perceived helpfulness), and (3) deliberation (speaking opportunities, adoption of a societal perspective on the issue, reasoned justification of ideas, and participant satisfaction).

**Results:**

We recruited 24 participants who reflected the diversity of views and life experiences of citizens of AAn living in Georgia. The expert testimony development process we undertook for creating balanced factual information was endorsed by experts' feedback. Deliberation process evaluation showed that while participation varied (average number of statements = 24, range: 3–62), all participants contributed. Participants were able to apply expert information and take a societal perspective to deliberate on the pros and cons of targeting individuals of AAn for HBOC screening in Georgia.

**Conclusions:**

The rigorous process of public engagement using deliberative democracy approach can successfully engage a citizenry with diverse and well-informed views, do so in a relatively short time frame and yield perspectives based on high quality discussion.

## Introduction

Obtaining public input and involvement in health service planning and delivery, and in setting health policy priorities is both critically needed and difficult to achieve (1). Strategies used to engage public participation span a continuum, ranging from discrete opportunities for engagement (e.g., focus groups, surveys) to serialized involvement requiring extended time commitments (e.g., coalitions, citizen science, or public hearings) (2). Indeed, the latter approaches require sustained interactions with citizens who can thoughtfully advise strategic decision making and the direction of public policy at the local or national level (3).

What public engagement looks like across this continuum differs considerably based on setting. Oftentimes, little attention is given to the appropriateness and standards of the methods used (4). As a result, approaches for public involvement proliferate with little systematic evidence regarding the quality of these approaches. Moreover, strategies to inform priority setting in public health contexts have been focused at the discrete end of the continuum. While discrete approaches benefit from being feasible, low cost, and less time demanding, these approaches arguably do not enable citizen participants to provide well-informed input.

Public engagement has particular importance in the case of complex health topics that involve new or controversial advances, where health priority setting requires balancing multiple tradeoffs. Input from members of the public may be especially helpful, when there is a sizable gap in scientific and public knowledge. Public engagement offers a process of involving target audiences as “co-creators” who can provide citizen perspectives on complex topics such as emerging genomic discoveries and related priorities. In turn, this approach can maximize the likelihood that programs and policies will be relevant, successful, and acceptable (5). Indeed, a recent systematic review examining public involvement in genomics research and translation suggested that sustainable, ongoing deliberative approaches to public participation should receive more attention (6).

Democratic deliberation (DD) is a public engagement strategy that has been used in numerous health contexts internationally (7–9). DD refers to a collective deliberation process that is conducted rationally and fairly among consumers (i.e., those with a stake in the issue at hand) and citizens (i.e., those who have no stake in the issue) (10). Unlike focus groups and other discrete methods, citizens and consumers are provided with focused and neutral factual information about the topic via “expert testimony”; participants' are encouraged to voice differing viewpoints, interests, and experiences; and groups deliberate about tradeoffs they view to be important to come to a consensus opinion that, in theory, would maximize the common good.

Previous literature has found that DD methods provide more authentic public opinions (11). Moreover, DD may be particularly useful when considering policies and programs for marginalized populations (11, 12). Enlisting these groups to generate and thoughtfully consider potential pros and cons of health policies and programs through the lens of personally experienced disparities can be an act of empowerment (12).

DD approaches are appropriate but have yet to be applied for public engagement in considering advances in genomic research and translation. Several national organizations concur that population screening to identify individuals and families at highest risk for inherited cancer syndromes is warranted (13–18). Low-cost genetic risk screening tools, such as family history screening, are available for several inherited cancer syndromes including heredity breast and ovarian cancer (HBOC) (13, 19). Women at increased risk of HBOC can be referred to genetic counseling, and if appropriate, genetic testing to inform lifesaving prevention and treatment options (20). Nonetheless, evidence suggests that early translation efforts to get these cancer genetic services in the hands of underrepresented minority populations are not overcoming existing disparity propensities. This is particularly concerning for women of African Ancestry (AAn) who are more likely to develop and die from aggressive breast cancers than women from other ancestry groups (with the exception of women of Ashkenazi Jewish ancestry) (21, 22). A growing number of studies also show that women of AAn are significantly less likely to seek cancer genetic services than other women even when receiving care in high-resourced specialty clinics (23–27). There have been numerous qualitative and quantitative studies to shed light on logistical and psychosocial barriers to genetic service uptake and research participation among minority populations (28–31). Yet efforts to address these barriers have not shown consistent improvement in uptake of cancer genetic services (30, 31).

Targeting women of AAn for HBOC screening could be controversial as it requires balancing multiple tradeoffs. A number of current realities add complexity to this consideration that warrant community deliberation: (1) deficiencies in family history-based genetic risk screening precision for those of AAn due to their low inclusion in HBOC basic science, treatment, and prevention research (32); (2) high rates of variants of uncertain significance and novel deleterious mutations among those of AAn due to the cascade of low access, provider referral to and uptake of testing (33, 34); (3) poorer understanding and acceptance of negative HBOC screening results (not at increased genetic risk) among those of AAn compared to Whites (35, 36); and (4) historic distrust of health care systems creating heightened privacy concerns related to genetic testing among AAn communities (37). Most research has focused on existing service delivery strategies (e.g., activated providers, telegenetics (27, 38). However, research has yet to enlist communities of AAn to thoughtfully consider whether targeted screening efforts is in the interest of the common good and warranted to redress their poor cancer outcomes.

**Figure 1:**
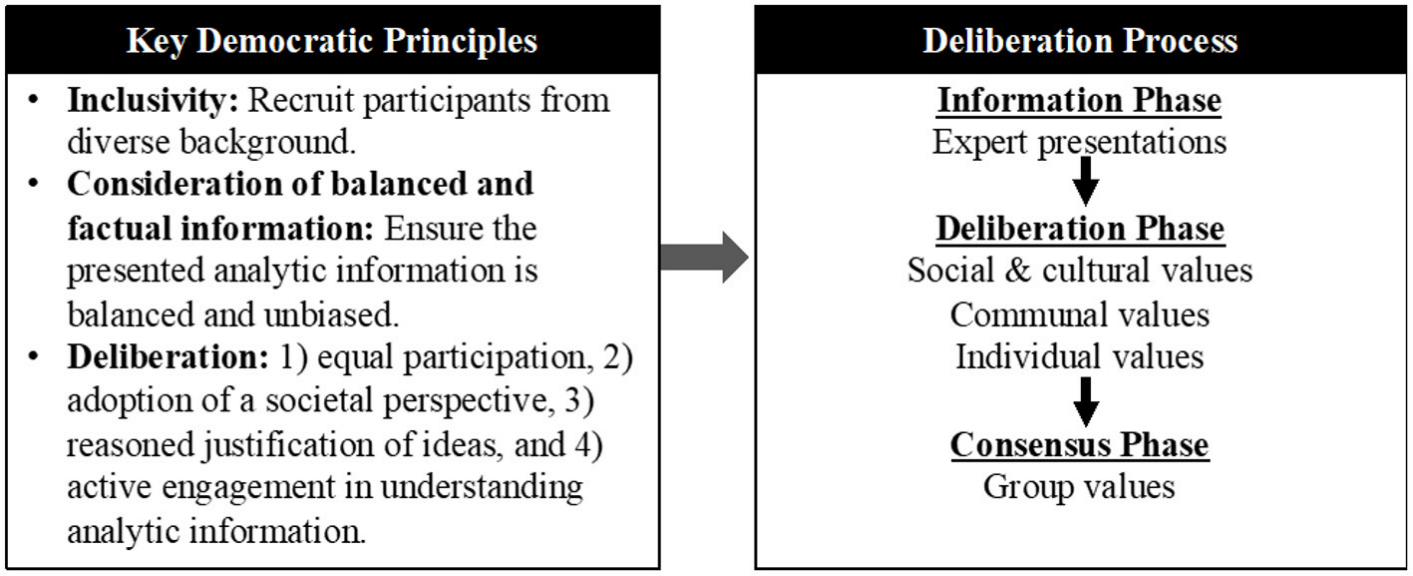
Conceptual framework of democratic deliberation design.

Because DD requires extensive researcher and community member investment, the feasibility of this method and whether it can achieve thoughtful and useful community input is unclear. To this end, we conducted a DD conference to gain community perspectives on targeting communities of AAn in Georgia for HBOC screening. Informed by previous research (39, 40), we considered three key democratic principles (Figure 1).

### Consideration of balanced and factual information

DD requires that participants have basic and unbiased understanding of the issues and tradeoffs to enable active discussion of the questions being deliberated. The requirement is that factual information be presented as free as possible from distortions or attempts at persuasion.

### Inclusivity

The deliberation group should reflect on the diversity of citizen and consumer views and life experiences (in this case citizens of AAn living in Southwest Georgia). Deliberation cannot be fully democratic if some parts of society are marginalized or excluded.

### Deliberation

Critical to optimal DD processes is that citizens discuss and weigh differing, and often competing, social values to reach consensus as a group (41). Members must have equal opportunity to take part in the discussion and deliberate, which involves listening and reflecting on others' perspectives before reaching conclusions. Members are encouraged to adopt a societal perspective on the issue in question, where the deliberation focuses on what is best for society, rather than on what is best for individual participants. In addition, the group reflects on what they hear and provides their rationale when offering comments.

For this manuscript, we aimed to describe: (1) a systematic process to create expert testimony materials that are informative, balanced and unbiased, (2) a multi-step process to recruit an inclusive group of participants who could reflect diversity of AAn in Southwest Georgia for a multi-day DD conference, and (3) a high-quality deliberation process characterized by participants having equal opportunity to contribute, active engagement in understanding presented information, adopting a societal perspective, and using reasoned justification to support their opinions.

## Methods

### Expert testimony development process

Participants gained understanding of the different scientific and ethical viewpoints, interests and experiences related to HBOC population screening. Development and formatting of expert testimony was a key design feature for enabling nuanced knowledge and understanding of the topic at hand. Experts in the areas of HBOC, population screening, and bioethics were members of the study team (Drs. McBride, Guan, McCullough and Dickert). These individuals conceptualized a short-list of topics they regarded to be essential for citizens to be able to thoughtfully consider the overarching issue. The testimony scripts and visual presentation were aligned with frameworks of health literacy and co-cultural communication theory (42) to circumvent the limitations imposed by low genomic literacy. Leveraging feedback from a meeting with stakeholders in Southwest Georgia, and in collaboration with our topical expert co-investigators (Drs. Gornick, Guan, McBride, McCullough, Dickert, Woods-Jaeger), we finalized audio-recorded PowerPoint testimony presentations that were 5–10 min long for the five topics relative to specific deliberation questions: *What is HBOC? Why is it important and how to identify people at risk for HBOC? Current HBOC screening program in Georgia, Why screen at the population level? What is ancestry and why African ancestry?* A single narrator was chosen for standardization and to maintain a neutral tone to the information presented. In total, participants viewed seven pre-recorded expert testimonies (two on day 1, three on day 2, and two on day 3).

### Population and recruitment

Our target population included citizens of AAn who were living in the surrounds of Albany, Georgia (182 miles South of Atlanta), the location of our community partnering organization Horizons Community Solutions (horizonscommunity.org; previously named Cancer Coalition of South Georgia). The population of Albany is estimated at 77,434; 72% of residents identify as having AAn; the Southwest region has 44.6% residents with AAn. A recent evaluation of HBOC screening in Albany's public health district shows that < 3% of women have completed family history-based screening that is provided by the Public Health Clinics in the area (43, 44).

In collaboration with our community partnering organization, working with the community for over 30 years, the study team developed a detailed recruitment rubric (Appendices 1, 2) to track and organize community partner- and participant-level information. We conducted brainstorming sessions to generate the full scope of constituencies of potential residents of the Albany area to ensure that an inclusive participant population was being reached. We organized these indicators of diversity along two domains: viewpoint diversity (e.g., age, gender, faith community involvement, cancer history) and having prior experiences that required consideration of the common good (e.g., civic engagement, community leadership experience, jury duty). Rationale of indicator selection is described in Appendix 2: Definitions/Rationale for Recruitment Rubric.

Based on these discussions, potential participants were required to: self-identify as African American/Black or Bi-racial, indicating African ancestry; and be ages 25 or older when risk-reducing interventions for *BRCA* mutation carriers are typically recommended to begin (45). Additionally, participants were required to speak English as all materials for the conference were created in English. Due to the COVID-19 pandemic, the conference was planned to be virtual requiring that participants have some comfort with the internet.

Recruitment took place in two phases. In the first phase, Horizon Community Solutions' network was leveraged to contact organizations and community-involved individuals to identify partners who might assist in sharing information about the project entitled “The Southwest Georgia Community Council on Hereditary Breast and Ovarian Cancer Citizen Discussion Group.” Identified community partners were sent a flier branded with the study name and were encouraged to share the information among their constituents. In the second phase, individuals who were interested in the study followed a link found on the study flier to complete a brief screener to assess basic eligibility criteria and indicators of viewpoint diversity (Appendix 3: Screener 1). Individuals who expressed continued interest in participating were then contacted via telephone by Horizon's staff for further screening on indicators suggestive of ability to consider the common good (Appendix 4: Screener 2).

Invitations to participate in the study were determined based on eligibility criteria and the individual's representing a key constituency identified in the rubric (i.e., viewpoint diversity and ability to consider common good). Participant enrollment was monitored weekly at a minimum to gauge representation of recruited participants and adjust the recruitment strategy as needed. Individuals who were invited and agreed to participate in the study consented via email before the sessions began. To further ensure feasibility of participation, technical assistance for using the online platform was also available for participants.

### Deliberation conference procedures

The research team assigned participants to five small groups prior to the discussion sessions, with the goal to have diverse constituencies represented within each small group. A trained facilitator moderated the discussion in each group. Facilitators were recruited from the community and had a background in public health, health education, or qualitative interviewing. Facilitators received a training workbook and 6-h of online deliberation training from a study team investigator (MCG) with expertise in qualitative research and in the conduct of DD sessions. Training materials and procedures were adapted from other published studies using this methodology (46). Facilitators were trained to engage participants with different learning and communication styles and allow the views of less vocal participants to be included (47). In particular, facilitators worked to ensure that everyone in their group understood the deliberation task and had the opportunity to speak and contribute, and that all the perspectives were heard and considered by the group. Facilitators also kept the discussions on topic and ensured each task was completed within the time available. Facilitators were trained to focus on the structure and process of the discussions, rather than content. Facilitators were instructed that they should not express any views on the matters under discussion, nor serve as sources of knowledge.

Participants were assigned to groups so as to balance the number of males and females, age, education-level distributions, and zip code. Participants remained in the same small group throughout all discussion sessions. Upon completion of the 2.5-day conference, participants were compensated with a total incentive of $200. All study activities were approved by Emory University IRB (IRB00114524).

Consistent with prior studies (1), ~ 1 week prior to the deliberation conference, discussion participants received a workbook by mail that included the meeting agenda, deliberation questions, guidelines for engaging and participating, and slides to be presented in the expert testimonies. The workbook also included activities, space for notes, and reflections that occurred during the deliberation. The deliberation conference included three Zoom sessions: a brief 75-min orientation meeting on Friday, March 12, and two 3.5-h sessions on Saturdays, March 13 and March 20. Deliberation involved viewing seven pre-recorded expert testimonies (two on day 1, three on day 2, and two on day 3) followed by generating and prioritizing pros and cons related to the question with group members. These discussions culminated in participants voting on whether or not they believed (DD Question 1) Georgia should continue its current way of identifying women at risk for HBOC and (DD Question 2) if Georgia should target all individuals of African ancestry in order to identify those at risk for HOBC.

### Data collection

#### Opportunity to consider balanced and factual information

The DD evaluation measures and sources of data are shown in Table 1. Using 10-point rating scales, we also asked DD participants how *helpful* the expert testimonies and interactions with peers and study team members was in their group discussions. Following completion of the DD conference, we convened a group of 14 stakeholders for a 2-h meeting to describe our DD process and hear their viewpoints on pros and cons of targeted screening and share citizen findings. Participants included community partners in Southwest Georgia (DB, JK), policy stakeholders who work across the state of Georgia and are involved in priority setting and decision making for cancer control activities. As part of the meeting, stakeholders viewed the expert testimonies and were asked to provide feedback regarding the perceived impartiality of the expert testimonies.

**Table 1:** Deliberation evaluation measures and data sources.

**Democratic principles**	**Measures**	**Data sources**
1. Inclusivity	– Age, gender, education, cancer history, employment status, church membership, experiences in voting in elections, serving on community committees	Recruitment screener 1 & 2
2. Opportunity to consider balanced and factual information	– Feedback on expert testimony scripts and videos	Project progress report
	– Perceived helpfulness	Participant post-deliberation survey
3. Deliberation	– Overall satisfaction – Willingness to participate in future deliberations	Participant post-deliberation survey
	– Equal participation – Active engagement to understand analytic information – Adoption societal perspective – Reasoned justification of ideas	Small group deliberation audio recordings

#### Inclusivity

Guided by the recruitment rubric, a database was created to record the number of individuals who: completed the initial online screener, were contacted for a second-round telephone interview, were deemed eligible, consented to participate, and attended each day of the conference. Indicators of viewpoint diversity included age, gender, education level, zip code, employment status (including retired), cancer history, faith community membership (Appendix 3: Screener 1). Indicators of ability to consider the common good (e.g., civic engagement, jury service, community leadership experience) were informally assessed during telephone interview (Appendix 4: Screener 2).

#### Deliberation

We coded deliberation session transcripts for four indicators of deliberation process quality (48): *speaking opportunities, adoption of a societal perspective on the issue in question, reasoned justification of ideas, and active engagement in understanding presented information*. We assessed *speaking opportunities* quantitatively by counting both the number of statements made by each participant, and the percentage of the statements each participant made in the deliberation (using the total number of statements made by all participants within the same small deliberation group as the denominator). These two measures represent differences in overall levels of participation - some participants provided many short statements, while others provided fewer, longer statements. Appendix 5 shows code definitions and quote examples. *Adoption of a societal perspective* was indicated when participants raised a pro or con based on group-level benefit or harm, or considered the issue from the perspective of cost to a social group. *Reasoned justification of ideas* was indicated when participants explained their viewpoint based on information raised in the expert testimonies, or when their comments indicated they were considering both sides of the issue. *Active engagement in understanding presented information* included statements indicating a participant was seeking to understand the information they had been given, such as confirming their understanding, clarifying a point that was made, checking for accuracy of their interpretation, and showing agreement with peers.

We also collected survey data on Day 2 regarding participants' experience of the process as a complement to the observational data on deliberation process quality. Survey items were adapted from those used previously (48) using 10-point rating scales (1 not at all to 10 very much). Example questions included, “*do you feel that your opinions were respected by your group,” “do you feel that the process that led to your group's responses was fair,”* and “*if given the opportunity, would you participate in a similar deliberation activity again.”*

### Data analysis

Quantitative descriptive analyses of survey data were conducted using SAS to characterize the participant's demographics and ratings of their experience with deliberation. Qualitative analyses of deliberation transcripts were conducted using MAXQDA. We adapted a qualitative coding scheme used by others to examine the deliberation process (48, 49), and we developed new codes based on careful reading of the transcripts and study team discussion (Appendix 5). Two study team members (YG and MCG) read through all transcripts and other team members read a subset of the transcripts (CM, JK, DB, SP). All study team members (*n* = 8) coded one small group session to ensure accuracy of coding, as well as to ensure the clarity and completeness of the coding scheme. Coding was then conducted by four team members (KS, GF, MCG, SP). After coding was completed, each transcript was systematically reviewed for the most commonly occurring themes and representative quotes were identified.

## Results

### Consideration of balanced and factual information

Post deliberation survey responses indicated that participants found the expert testimony videos very useful in their deliberations (M = 9.29, SD = 1.52, range =1–10), and reported it was very helpful to have the opportunity to discuss the issues with other participants (M = 9.43, SD = 1.73, range=1–10). Feedback from community and policy stakeholders supported that the videos presented balanced factual information (e.g., what genetic testing can and cannot tell you) without pushing any agenda or being persuasive.

### Feasibility of recruiting an inclusive citizen group

Horizons Community Solutions contacted 149 community partners to facilitate recruitment. The community partners circulated study fliers to their constituents, reaching 23 counties and 20 zip codes in Southwest GA. Across these counties, 78 individuals (59 females, 19 males) completed the online screener and were interested in participating in the online citizen discussion group. Horizon Community Solutions staff then conducted 45 second-round interviews with interested individuals and filled out the recruitment rubric to further assess eligibility criteria; 31 were selected and consented to participate in the citizen discussion group.

**Figure 2:**
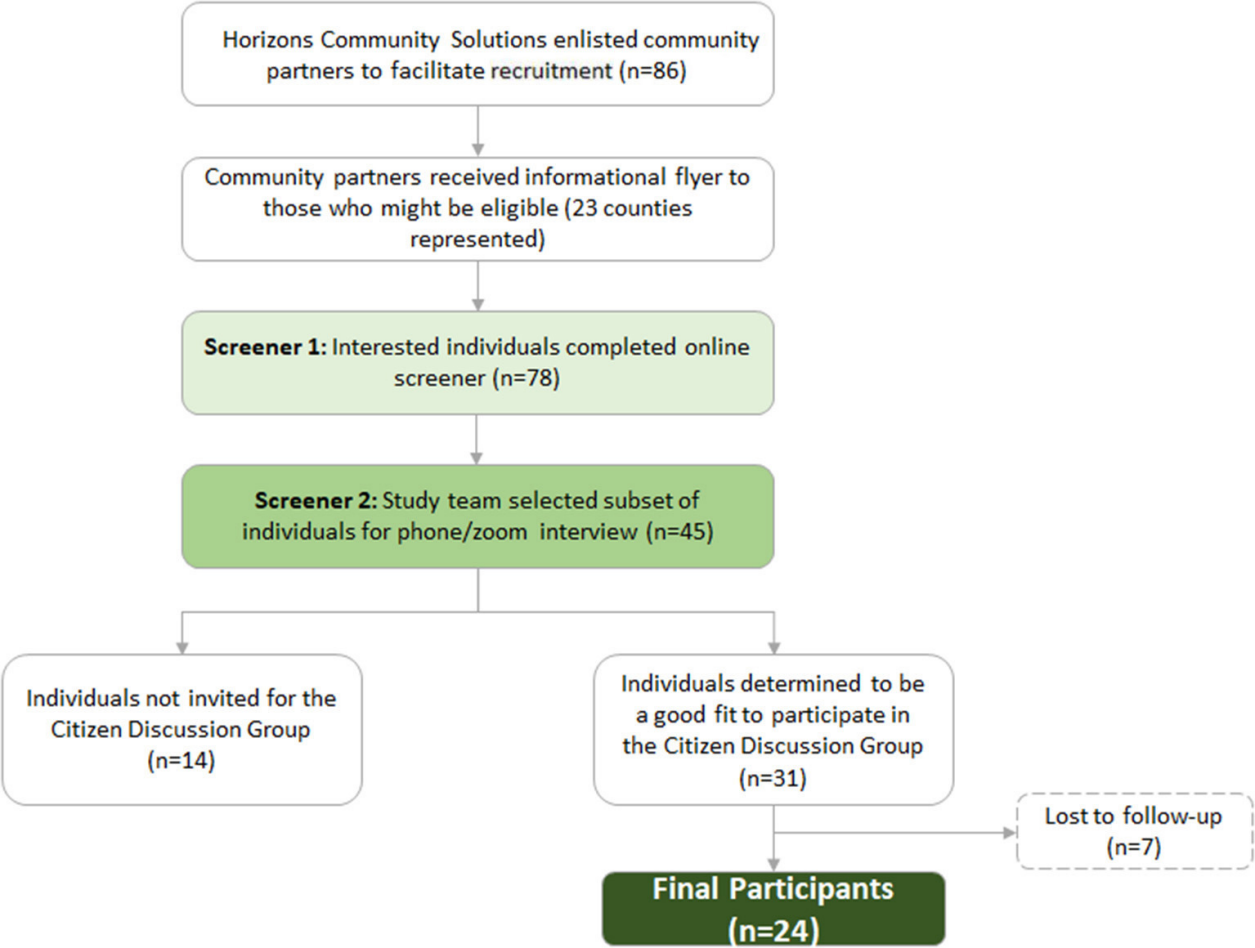
Recruitment flow diagram.

Only participants who attended both days of the conference and completed all post-conference surveys are included in the final sample (Table 2). Seven participants (22.5%) were lost to follow-up as 26 individuals attended Day 1 of the conference, and 24 attended Day 2 (one participant did not return, and one participant was asked not to attend Day 2 due to lack of engagement). All participants had Internet access at home. Most participants used email daily (*n* = 21, 87.5%) and video conferenced monthly or more frequently (*n* = 21, 87.5%). See Figure 2 for a recruitment flow diagram.

**Table 2:** Participant characteristics reflecting viewpoints diversity (*N* = 24).

**Member characteristics**	**Total (*N* = 24)**
**Gender**	
Female	19 (79.2%)
**Age**	
20–29	2 (8.3%)
30–39	2 (8.3%)
40–49	9 (37.5%)
50–59	6 (25%)
60–69	5 (20.8%)
**Education**	
High school graduate	2 (8.4%)
Some college	5 (20.8%)
College graduate	8 (33.3%)
Trade school	2 (8.3%)
Postgraduate work	7 (29.2%)
Employment status^*^	
Unemployed/Self-employed	5 (21.7%)
Employed	11 (47.8%)
Retired	7 (30.4%)
**Healthcare professional** (yes)	
**Time living in SWGA**	3 (12.5%)
>1 year	1 (4.2%)
1–5 years	2 (8.3%)
More than 5 years	21 (87.5%)
**Breast cancer dx** (yes)	1 (4.2%)
**Primary care in FQHC** (yes)	7 (38.9%)
**Member of church** (yes)	20 (83.3%)

* 1 missing response.

Most participants were long term residents of Southwest Georgia (88%). Participants ranged in age from 27 to 66 (mean 48.7, SD = 11.6). Just over half of the participants (55%) had some college or were a college graduate, 12% had some high school or were a high school graduates and two participants attended trade school. Seven participants were retired at the time of the conference, 12% were unemployed, and 54% were employed including two participants who were self-employed. One third received federally qualified health care (39%). Most had some faith involvement, however, 16% were not members of a church. One participant reported a previous breast cancer diagnosis. In addition, 21% of participants were men (*n* = 5).

### Deliberation process quality

#### Equal participation

We found considerable variation in the level of participation in all five groups. Statement counts for each participant were calculated by tallying each time the individual meaningfully contributed (i.e. exclusion of comments lacking substantive content; for example, passive agreement, comments not related to the discussion) during the deliberation question discussion. The number of statements by participants ranged from a low of 3 to a high of 62 (average = 24). The degree to which individual participants contributed to the discussion varied from 3 to 24% of total statements made during deliberation. Participants were more active and made more statements in Groups 3–5, where facilitators were more involved in actively moderating the discussion, with many checking and paraphrasing statements.

#### Active engagement in understanding presented information

Review of the transcripts suggested that participants sought to understand the expert testimonies they had been given. For example, participants often re-stated or reflected back information that they had heard from another member to check for accuracy of information, or to confirm a shared understanding of the facts or issues being discussed – e.g., “*I wanted to ask a question, only one percent of those people – was that ‘had the BRCA gene?' Is that what it said?” “It's more so asking the question of to what degree of genetic similarity is required to be considered of African ancestry. Because my skin might not reflect that. My recent family history might not reflect that.”* These statements illustrate how participants attempted to analyze the information presented to come to a correct understanding.

Another example of clarifying understanding was when participants showed agreement or disagreement with their peers, or referred to statements made by their peers. Overall, agreement with peers occurred more frequently than disagreement. For example, one participant endorsed her group member's views on the cons of genetic screening: “*I agree with what he stated about the insurance companies using that information to either deny insurance or give inflated prices.”*

#### Reasoned justification of ideas

Engaging in quality deliberation is indicated when participants show willingness to explain their own views, rather than just asserting them. For examples, participants often referred to expert testimonies to justify their reasoning for a “pro” or “con” that they were asserting. In all five groups we found that participants recalled and referred to facts from expert testimony presentations in their deliberations. For example, one participant recalled information from an expert testimony about family history screening yielding false negative results as a rationale for an asserted con related to targeting HBOC screening to those of AAn: “*If it's a false negative, you could lose the benefit of treatment early on because you think you're okay. I was saying the uncertainty of the screening results was definitely a top con.”* Participants also referred to concepts presented in the expert testimonies to justify their views using terminology related to genetics and inheritance, and the difference between family history and ancestry. For example, “*From the video, it showed to me that the African American descent had more of a possibility of having breast cancer and ovarian cancer than any other ethnicity.”*

Participants' ability to consider both sides of an issue by offering a counterpoint to a pro or con was also indicative of reasoned justification. For example, one participant indicated, “*Part of that is going to be a pro. Part of that is going to be a con. The positive part is that now we know they need treatment. The next step will be, ‘Now, how do we find that treatment? How do we get them into the treatment? Can they afford it? Is it even available in that community?' All that ripples after that.”* This skill was observed infrequently during the deliberation.

#### Adoption of a societal perspective

Adopting a societal perspective was indicated when participants gave voice to a group perspective that deviated from their own personal interests. For example, a participant raised a pro based on group-level benefit: “*Even in our community, I think that all genders and races can benefit from it because this is a low-income area here that we're living in.”*

Participants also demonstrated the ability to consider pros and cons of targeting the screening program among AAn communities from the perspective of economic costs to society. Here a participant considered the pro of targeting communities of AAn: “*Prevention and targeting prevention is less expensive than chemo, radiation, or hospitalization. So, by focusing on prevention, we can help cut down on healthcare costs, which is a plus for everybody across the board.”* Another participant was concerned about expanding the screening program considering potential insurance discrimination: “*The insurance would come into play at some point where they may want to charge higher premiums for someone who does have that hereditary factor.”*

However, we also found several instances of participants offering views indicative of their personal interests or experiences. Here a participant is considering their own race/ethnic makeup as a justification for why they believe everyone should be screened: “*I just found out about a week or so ago I was 20 percent Mawi or something. I don't even know what that is so. We just don't know. So, you might look like you might be a certain race and may not be 100 percent that race. So, I think everybody should be screened.”* Another participant reflected on their own experiences: “*This part is kinda tough because I also like the idea of – where I have issue is no consistency. And I have been doing my mammogram. I have masses in my breasts that they've taken out, and some they decided not to take out. But nobody has said to me, “Hey, do you wanna test for any genetic problems?”* However, participants' justifications based on personal interests were relatively less common (*n* = 25) compared to statements reflecting community interests (*n* = 134).

#### Satisfaction with deliberation process

Participants viewed the community deliberation process to be positive. Participants felt their opinions were respected (M = 8.86, SD = 0.47, range =1–10), they were listened to by the facilitator (M = 8.82, SD = 0.59, range=1–10), the discussion process was fair (M = 8.82, SD = 0.85, range=1–10), and they were willing to abide by the policy decision put forth by the group even if they held a different opinion (M = 8.82, SD = 0.50, range=1–10). Most participants (*N* =18, 79%) indicated that they would be willing to participate if a conference was held in-person instead of online. When asked if they would participate in a similar conference (i.e., online) on another topic, all but two participants (92%) said they would be willing or very willing.

## Discussion

Efforts to foster public engagement in health promotion interventions and policy design have focused largely on the low end of the engagement continuum. For example, focus groups and structured interviews predominate as public engagement strategies. These methods commonly garner participants' personal views, experiences and preferences relating to a health topic drawing from small and self-selected samples (50, 51). Information processing theories suggest these approaches likely do not motivate participants to do the work of intentional reflection, consider the complexities of new information, or feel culturally empowered to believe that their viewpoints can make a difference (52–55). This may be especially limiting in guiding interventions and policies in complex health contexts that are unfamiliar to target audiences.

Engagement strategies higher on the continuum such as deliberative democracy (56) that require more active and ongoing citizen engagement (e.g., becoming informed about the topic, learning about ethical concepts such as the common good, and repeated peer discussion) are uncommon as they require more effort and resources to accomplish (8). Implementing deliberative democracy and other high-end engagement approaches may be challenging but worth the effort particularly for timely yet novel genomics policy issues if: (1) a diversity of well-informed citizen views can be attained; and (2) the outcomes can be shown to be of higher quality than approaches lower on the engagement continuum (57). Our results suggest that with careful and diligent methodology, a deliberative democracy approach can successfully engage a citizenry with diverse and well-informed views, do so in a relatively short time frame and yield perspectives based on high quality discussion. We based these conclusions on three democratic principles (i.e., inclusivity, opportunity to consider factual information free of distortion, and deliberation).

First, we were able to recruit an inclusive group of citizens of African ancestry. This thoughtful and focused recruitment process enabled citizens often excluded from public health policy decision making to participate in genomic research in accordance with their communities' values and priorities (58). Recruitment efforts were facilitated by strong collaborations with local community organizations and their social networks. Community partners suggested characteristics specific to their area that would indicate viewpoint diversity (e.g., age, gender, faith community involvement, cancer history) and experiences that required consideration of the common good (e.g., civic engagement, community leadership experience, jury duty). We used these indicators to vet our participants through a structured interview process to create viewpoint diversity amongst our participants that, in turn, would encourage a well-rounded discussion centered on the common good.

We structured the expert testimonies, print materials and deliberation sessions to promote understanding of relevant information, skills to use in discussions with peers, and how to build consensus. All participants viewed the same information describing advantages and disadvantages of specifically targeting communities of African ancestry for family history-based screening for HBOC risk. Participants rated the expert testimony content as concise, unbiased and helpful in their deliberation.

Several studies have attempted to establish hierarchies that rank various levels of public involvement in health care decision making in an attempt to measure quality of a deliberation. Arnstein's (59) original work categorizing citizen participation presented an “eight rung ladder,” however several more recent studies suggest simpler frameworks. For example, the five degrees of participation: informing, consultation, partnership, delegated power, and citizen control (60). Another framework, specifically developed to guide genomics activities, uses four themes for deliberative reflection: fairness, context, heterogeneity, and recognizing tensions and conflict (61). Congruently, we found that participants' deliberation met these and other previously identified quality frameworks (48, 62, 63). Dissimilarly, the current study focused on aspects of quality specific to public policy such as adoption of a societal perspective or “the common good.” This is the idea that what is best for the individual is not always what is best for the larger community. This concept is critical when discussing and setting priorities for public health policy, as it impacts the entire community, not just the individual participating in the peer deliberation.

Our analyses of transcripts suggest that there was active participation in which individuals were heard and respected. While we observed significant variations between participants where some participants spoke more than others, we attribute this to differences in styles with some participants expressing their views more concisely than others. In post-deliberation surveys, participants also strongly endorsed feeling able to participate, respected and heard.

We examined other process evaluation indicators to assess whether the deliberation process succeeded in encouraging citizens considered expert testimonies to justify their input. Indeed, we found that participants justified their viewpoints by referring to information they learned from the expert testimonies, previous knowledge of the subject, and/or showing agreement with comments made by other participants. However, this did not occur consistently suggesting that additional brief training of citizens in how to support their viewpoints in discussions with fellow participants could be helpful.

Consistent with taking the perspective of the common good, citizens gave thoughtful and expansive consideration of the pros and cons of targeting those of African ancestry for accelerated HBOC screening in Southwest Georgia. Our citizen participants generated a more diverse slate of pros and cons than state-level cancer policy stakeholders. As a follow-up to the community deliberation, the study team shared the citizen generated advantages and disadvantages with state cancer policy stakeholders in Georgia. Stakeholders not only supported participants' viewpoints but also complimented how unique and useful citizen perspectives would be for setting related cancer policy priorities in the state.

In conclusion, while a deliberative approach might be considered resource intensive, the community partnerships, recruitment efforts, and facilitator training efforts we employed led to high quality public input. Recruiting for and implementing less intensive approaches such as focus groups can be demanding. An important consideration is whether the quality of the information attained is worth the effort. Yet, few public engagement studies have evaluated the quality of the information yielded by less intensive engagement strategies. Much of the extra effort we expended was in developing the recruitment rubric and preparing the participants to thoughtfully reflect on the issues in family history screening. Recruitment rubrics are often implemented when forming community advisory boards. Indeed, these boards could serve as an ongoing group engaged for deliberation. The unbiased expert testimonies were regarded as critically important for reflective participation in intervention and policy development. Once the testimonies have been developed, however, these materials would only require periodic updating similar to most health education materials. It is noteworthy that we were able to complete all these steps in a 9-month time frame.

Like any study, there are limitations to our process. We relied on self-determination of African ancestry for the current study. We acknowledge that it is currently not possible to determine or differentiate African ancestry from a person who identifies as being African American in the absence of a genetic test. Although we used a multi-step and systematic process for developing expert testimonies, we did not conduct a formal evaluation of how the information was perceived by participants. Further, our process to assess feasibility of the method was conducted in one geographic area and relating to one health context. The process likely would need to be adapted for other community settings and health contexts.

In sum, we conducted a rigorous process of public engagement using deliberative democracy techniques, showed it to be feasible and to yield high quality output. This and other public engagement methods warrant more attention. This can begin by challenging ourselves to operationalize higher intensity strategies to ensure that our interventions and policies align with citizen perspectives. Ultimately, this pursuit has the strongest likelihood for public health benefits.

## Data availability statement

The original contributions presented in the study are included in the article/Supplementary material, further inquiries can be directed to the corresponding author.

## Ethics statement

The studies involving human participants were reviewed and approved by Emory University IRB. Written informed consent for participation was not required for this study in accordance with the national legislation and the institutional requirements.

## Author contributions

All authors conceptualized and designed the study, prepared the manuscript, analyzed and interpreted data, and critically revised the manuscript.

## Funding

This work was funded by the National Cancer Institute (R21CA238356).

## Conflict of interest

The authors declare that the research was conducted in the absence of any commercial or financial relationships that could be construed as a potential conflict of interest.

## Publisher's note

All claims expressed in this article are solely those of the authors and do not necessarily represent those of their affiliated organizations, or those of the publisher, the editors and the reviewers. Any product that may be evaluated in this article, or claim that may be made by its manufacturer, is not guaranteed or endorsed by the publisher.

## References

[1] Mitton C Smith N Peacock S Evoy B Abelson J. Public participation in health care priority setting: a scoping review. Health Policy. (2009) 91:219–28. 10.1016/j.healthpol.2009.01.005 19261347

[2] Kitzhaber JA. Prioritising health services in an era of limits: the Oregon experience. BMJ. (1993) 307:373–7. 10.1136/bmj.307.6900.373 8374424 PMC1678226

[3] Florin D Dixon J. Public involvement in health care. BMJ. (2004) 328:159–61. 10.1136/bmj.328.7432.159 14726350 PMC314519

[4] Contandriopoulos D. A sociological perspective on public participation in health care. Soc Sci Med. (2004) 58:321–30. 10.1016/s0277-9536(03)00164-3 14604618

[5] Rychetnik L Carter SM Abelson J Thornton H Barratt A Entwistle VA. Enhancing citizen engagement in cancer screening through deliberative democracy. J Natl Cancer Inst. (2013) 105:380–6. 10.1093/jnci/djs649 23378639

[6] Wait S Nolte E. Public involvement policies in health: exploring their conceptual basis. Health Econ Policy Law. (2006) 1(Pt 2):149–62. 10.1017/S174413310500112X 18634687

[7] Abelson J Forest PG Eyles J Smith P Martin E Gauvin FP. Deliberations about deliberative methods: issues in the design and evaluation of public participation processes. Soc Sci Med. (2003) 57:239–51. 10.1016/s0277-9536(02)00343-x 12765705

[8] Gutmann A Thompson D. Why Deliberative Democracy? Princeton University Press; (2004). Available online at: https://press.princeton.edu/books/paperback/9780691120195/why-deliberative-democracy (accessed March 16, 2022).

[9] Abelson J Forest PG Eyles J Smith P Martin E Gauvin FP. Obtaining public input for health-systems decision-making: past experiences and future prospects. Canadian Public Administ. (2002) 45:70–97. 10.1111/j.1754-7121.2002.tb01074.x

[10] Benhabib S. Democracy and Difference: Contesting the Boundaries of the Political. Princeton University Press (1996).

[11] Sullivan G Cheney A Olson M Haynes T Bryant K Cottoms N. Rural African Americans' perspectives on mental health: Comparing focus groups and deliberative democracy forums. J Health Care Poor Underserved. (2017) 28:548–65. 10.1353/hpu.2017.0039 28239018

[12] Subica AM Brown BJ. Addressing health disparities through deliberative methods: citizens' panels for health equity. Am J Public Health. (2020) 110:166–73. 10.2105/AJPH.2019.305450 31855474 PMC6951363

[13] Moyer VA. US Preventive services task force risk assessment, genetic counseling, and genetic testing for BRCA-related cancer in women: us preventive services task force recommendation statement. Ann Intern Med. (2014) 160:271–81. 10.7326/M13-2747 24366376

[14] Force U. Risk Assessment, genetic counseling, and genetic testing for *BRCA*-Related cancer: recommendation statement. AFP. (2020) 101:233–8. 10.1001/jama.2019.10987 32053325

[15] Evaluation of Genomic Applications in Practice and Prevention (EGAPP) Working Group. Recommendations from the EGAPP working group: can tumor gene expression profiling improve outcomes in patients with breast cancer? Genet Med. (2009) 11:66–73. 10.1097/GIM.0b013e3181928f56 19125125 PMC2743614

[16] Hampel H Bennett RL Buchanan A Pearlman R Wiesner GL. Guideline development group, american college of medical genetics and genomics professional practice and guidelines committee and national society of genetic counselors practice guidelines committee. A practice guideline from the American college of medical genetics and genomics and the national society of genetic counselors: referral indications for cancer predisposition assessment. Genet Med. (2015) 17:70–87. 10.1038/gim.2014.147 25394175

[17] Hegde M Ferber M Mao R Samowitz W Ganguly A. Working group of the American college of medical genetics and genomics (ACMG) laboratory quality assurance committee. ACMG technical standards and guidelines for genetic testing for inherited colorectal cancer (Lynch syndrome, familial adenomatous polyposis, and MYH-associated polyposis). Genet Med. (2014) 16:101–16. 10.1038/gim.2013.166 24310308

[18] Maxwell KN Hart SN Vijai J Schrader KA Slavin TP Thomas T. Evaluation of ACMG-guideline-based variant classification of cancer susceptibility and non-cancer-associated genes in families affected by breast cancer. Am J Hum Genet. (2016) 98:801–17. 10.1016/j.ajhg.2016.02.024 27153395 PMC4863474

[19] Bellcross CA Kolor K Goddard KAB Coates RJ Reyes M Khoury MJ. Awareness and utilization of BRCA1/2 testing among U. S primary care physicians. Am J Prev Med. (2011) 40:61–6. 10.1016/j.amepre.2010.09.027 21146769

[20] Kuchenbaecker KB Hopper JL Barnes DR Phillips KA Mooij TM Roos-Blom M. Risks of breast, ovarian, and contralateral breast cancer for BRCA1 and BRCA2 mutation carriers. JAMA. (2017) 317:2402–16. 10.1001/jama.2017.7112 28632866

[21] Stark A Kleer CG Martin I Awuah B Nsiah-Asare A Takyi V. African ancestry and higher prevalence of triple-negative breast cancer: findings from an international study. Cancer. (2010) 116:4926–32. 10.1002/cncr.25276 20629078 PMC3138711

[22] Siegel RL Miller KD Fuchs HE Jemal A. Cancer Statistics, 2021. CA Cancer J Clin. (2021) 71:7–33. 10.3322/caac.21654 33433946

[23] Manrriquez E Chapman JS Mak J Blanco AM Chen LM. Disparities in genetics assessment for women with ovarian cancer: can we do better? Gynecol Oncol. (2018) 149:84–8. 10.1016/j.ygyno.2017.10.034 29605055

[24] Armstrong K Micco E Carney A Stopfer J Putt M. Racial differences in the use of BRCA1/2 testing among women with a family history of breast or ovarian cancer. JAMA. (2005) 293:1729–36. 10.1001/jama.293.14.1729 15827311

[25] Pagán JA Su D Li L Armstrong K Asch DA. Racial and ethnic disparities in awareness of genetic testing for cancer risk. Am J Prev Med. (2009) 37:524–30. 10.1016/j.amepre.2009.07.021 19944919

[26] Levy DE Byfield SD Comstock CB Garber JE Syngal S Crown WH. Underutilization of BRCA1/2 testing to guide breast cancer treatment: black and Hispanic women particularly at risk. Genet Med. (2011) 13:349–55. 10.1097/GIM.0b013e3182091ba4 21358336 PMC3604880

[27] Butrick M Kelly S Peshkin BN Luta G Nusbaum R Hooker GW. Disparities in uptake of BRCA1/2 genetic testing in a randomized trial of telephone counseling. Genet Med. (2015) 17:467–75. 10.1038/gim.2014.125 25232856 PMC4364924

[28] Halbert CH McDonald J Vadaparampil S Rice L Jefferson M. Conducting precision medicine research with African Americans. PLoS One. (2016) 11:e0154850. 10.1371/journal.pone.0154850 27441706 PMC4956119

[29] McDonald JA Barg FK Weathers B Guerra CE Troxel AB Domchek S. Understanding participation by African Americans in cancer genetics research. J Natl Med Assoc. (2012) 104:324–30. 10.1016/s0027-9684(15)30172-3 23092046 PMC3760677

[30] Allford A Qureshi N Barwell J Lewis C Kai J. What hinders minority ethnic access to cancer genetics services and what may help? Eur J Hum Genet. (2014) 22:866–74. 10.1038/ejhg.2013.257 24253862 PMC4060110

[31] Mathew SS Barwell J Khan N Lynch E Parker M Qureshi N. Inclusion of diverse populations in genomic research and health services: Genomix workshop report. J Community Genet. (2017) 8:267–73. 10.1007/s12687-017-0317-5 28755064 PMC5614885

[32] Bondy ML Newman LA. Breast cancer risk assessment models: applicability to African-American women. Cancer. (2003) 97:230–5. 10.1002/cncr.11018 12491486

[33] Flegal KM Graubard BI Williamson DF Gail MH. Cause-specific excess deaths associated with underweight, overweight, and obesity. JAMA. (2007) 298:2028–37. 10.1001/jama.298.17.2028 17986696

[34] Pal T Bonner D Kim J Monteiro AN Kessler L Royer R. Early onset breast cancer in a registry-based sample of African-American women: BRCA mutation prevalence, and other personal and system-level clinical characteristics. Breast J. (2013) 19:189–92. 10.1111/tbj.12083 23320992

[35] Guan Y Condit CM Escoffery C Bellcross CA McBride CM. Do women who receive a negative BRCA1/2 risk result understand the implications for breast cancer risk? Public Health Genomics. (2019) 22:102–9. 10.1159/000503129 31597139

[36] Foster C Watson M Eeles R Eccles D Ashley S Davidson R. Predictive genetic testing for BRCA1/2 in a UK clinical cohort: three-year follow-up. Br J Cancer. (2007) 96:718–24. 10.1038/sj.bjc.6603610 17285126 PMC2360079

[37] Adams LB Richmond J Corbie-Smith G Powell W. Medical mistrust and colorectal cancer screening among African Americans. J Community Health. (2017) 42:1044–61. 10.1007/s10900-017-0339-2 28439739 PMC5654700

[38] Bonevski B Randell M Paul C Chapman K Twyman L Bryant J. Reaching the hard-to-reach: a systematic review of strategies for improving health and medical research with socially disadvantaged groups. BMC Med Res Methodol. (2014) 14:1–29. 10.1186/1471-2288-14-42 24669751 PMC3974746

[39] O'Doherty KC Burgess MM Edwards K Gallagher RP Hawkins AK Kaye J. From consent to institutions: designing adaptive governance for genomic biobanks. Soc Sci Med. (2011) 73:367–74. 10.1016/j.socscimed.2011.05.046 21726926

[40] O'Doherty KC Hawkins AK Burgess MM. Involving citizens in the ethics of biobank research: informing institutional policy through structured public deliberation. Soc Sci Med. (2012) 75:1604–11. 10.1016/j.socscimed.2012.06.026 22867865

[41] Smith G Wales C. Citizens' Juries and Deliberative Democracy. Polit Stud. (2000) 48:51–65. 10.1111/1467-9248.00250

[42] Lapinski MK Orbe MP. Evidence for the construct validity and reliability of the co-cultural theory scales. Commun Methods Meas. (2007) 1:137–64. 10.1080/19312450701399388

[43] Traxler LB Martin ML Kerber AS Bellcross CA Crane BE Green V. Implementing a screening tool for identifying patients at risk for hereditary breast and ovarian cancer: a statewide initiative. Ann Surg Oncol. (2014) 21:3342–7. 10.1245/s10434-014-3921-1 25047474

[44] Georgia Cancer Screenings & Genetics Services. Available online at: https://www.georgiacancerinfo.org/screening-genetics/. (accessed May 6, 2021).

[45] U.S. Preventive Services Task Force. Genetic risk assessment and BRCA mutation testing for breast and ovarian cancer susceptibility: recommendation statement. Ann Intern Med. (2005) 143:355–61. 10.7326/0003-4819-143-5-200509060-00011 16144894

[46] Kim SYH Wall IF Stanczyk A De Vries R. Assessing the public's views in research ethics controversies: deliberative democracy and bioethics as natural allies. J Empir Res Hum Res Ethics. (2009) 4:3–16. 10.1525/jer.2009.4.4.3 19919315 PMC2853797

[47] O'Doherty K Gauvin FP Grogan C Friedman W. Implementing a public deliberative forum. Hastings Cent Rep. (2012) 42:20–3. 10.1002/hast.28 22733326

[48] De Vries R Stanczyk AE Ryan KA Kim SYH A. Framework for assessing the quality of democratic deliberation: enhancing deliberation as a tool for bioethics. J Empir Res Hum Res Ethics. (2011) 6:3–17. 10.1525/jer.2011.6.3.3 21931233 PMC3336203

[49] De Vries R Stanczyk A Wall IF Uhlmann R Damschroder LJ Kim SY. Assessing the quality of democratic deliberation: a case study of public deliberation on the ethics of surrogate consent for research. Soc Sci Med. (2010) 70:1896–903. 10.1016/j.socscimed.2010.02.031 20378225 PMC2866810

[50] Sanders Thompson VL Ackermann N Bauer KL Bowen DJ Goodman MS. Strategies of community engagement in research: definitions and classifications. Transl Behav Med. (2021) 11:441–51. 10.1093/tbm/ibaa042 32421173 PMC8135186

[51] O.Nyumba T Wilson K Derrick CJ Mukherjee N. The use of focus group discussion methodology: Insights from two decades of application in conservation. Methods in Ecology and Evolution. (2018) 9:20-32. 10.1111/2041-210X.12860

[52] Baumeister RF Newman LS. Self-regulation of cognitive inference and decision processes. Pers Soc Psychol Bull. (1994) 20:3–19. 10.1177/0146167294201001

[53] Fiske ST Taylor SE. Social Cognition. McGraw-Hill (1991).

[54] Kahneman D Slovic P Tversky A eds. Judgment under Uncertainty: Heuristics and Biases. Cambridge University Press (1982). 10.1017/CBO9780511809477 17835457

[55] Gallegos P. Choices, values, and frames. Am Psychol. (1983) 39:341–50.

[56] Ryfe D. Does deliberative democracy work? Annu Rev Polit Sci. (2005):8:49–71. 10.1146/annurev.polisci.8.032904.154633

[57] Bombard Y Abelson J Simeonov D Gauvin FP. Citizens' perspectives on personalized medicine: a qualitative public deliberation study. Eur J Hum Genet. (2013) 21:1197–201. 10.1038/ejhg.2012.300 23340511 PMC3798829

[58] Blacksher E Hiratsuka VY Blanchard JW Lund JR Reedy J Beans JA. Deliberations with American Indian and Alaska native people about the ethics of genomics: an adapted model of deliberation used with three tribal communities in the United States. AJOB Empir Bioeth. (2021) 12:164–78. 10.1080/23294515.2021.1925775 34125006 PMC8274345

[59] Arnstein S. A ladder of citizen participation. J Am Inst Plan. (1969) 35:216–24.

[60] Safaei J. Deliberative democracy in health care: current challenges and future prospects. J Healthc Leadersh. (2015) 7:123–36. 10.2147/JHL.S70021 29355181 PMC5740990

[61] Murtagh MJ Machirori M Gaff CL Blell MT de Vries J Doerr M. Engaged genomic science produces better and fairer outcomes: an engagement framework for engaging and involving participants, patients and publics in genomics research and healthcare implementation. Wellcome Open Res. (2021) 6:311. 10.12688/wellcomeopenres.17233.1 35592835 PMC9086526

[62] Goold SD Danis M Abelson J Gornick M Szymecko L Myers CD. Evaluating community deliberations about health research priorities. Health Expect. (2019) 22:772–84. 10.1111/hex.12931 31251446 PMC6737773

[63] Goold SD Neblo MA Kim SYH Vries RD Rowe G Muhlberger P. What is good public deliberation? Hastings Center Report. (2012) 42:24–6. 10.1002/hast.29 22733327

