# Supplementary material for: Testing a deliberative democracy method with citizens of African ancestry to weigh pros and cons of targeted screening for hereditary breast and ovarian cancer risk
Source: Front Public Health. 2022 Nov 8;10:984926. doi: 10.3389/fpubh.2022.984926 (PMC9679525; doi:10.3389/fpubh.2022.984926)
Supplement: Supplementary file 1 [file Table_1.DOCX]

**APPENDIX 1. Recruitment diversity rubric for in-person deliberation conference**

| **Viewpoint diversity** | **Ability to consider common good** | | | | | | | |
| --- | --- | --- | --- | --- | --- | --- | --- | --- |
|  | Vote in elections | | Served as a committee leader | | Served on a jury | | Advocacy experience | |
|  | + | - | + | - | + | - | + | - |
| Male gender |  |  |  |  |  |  |  |  |
| Younger than age 40 |  |  |  |  |  |  |  |  |
| Retired (+/-) |  |  |  |  |  |  |  |  |
| Tax payer, Employed at least part-time (+/-) |  |  |  |  |  |  |  |  |
| Consistent Church Membership & Attendance (+/-) |  |  |  |  |  |  |  |  |
| Receives care in Federally Qualified Healthcare Centers (+/-) |  |  |  |  |  |  |  |  |
| Low income census tracks (+/-) |  |  |  |  |  |  |  |  |
| Personal cancer history (+/-) |  |  |  |  |  |  |  |  |

*Not faith-based social clubs: Sororities, book clubs, etc.

**APPENDIX 2. Definitions/Rationale for Recruitment Rubric**

| **Viewpoint Diversity Items** | | |
| --- | --- | --- |
| **Item** | **Definition** | **Rationale** |
| **Consistent Church Membership & Attendance** | Individuals who ascribe to a faith tradition hold opinions that align with moral values. | Most of these individuals have been schooled in the tenets of their faith tradition and can share the beliefs that arise from the doctrines. (It would be important to represent different faith traditions if possible) |
| **Receives care in FQHCs** | Has experience of limited options for healthcare, low choices/poorly funded healthcare/uninsured. | People who have limited opportunities for healthcare services will have: a unique perspective regarding priorities, experiences with overwhelmed systems and limitations on resources. Example- health departments encouraging screening for cancer, but when found to be positive, treatment resources may not be available. |
| **Low income census tracks** | Any census tracks (or equivalent geographic area) in which at least 50% of households have an income less than 60% of the Area Median Gross Income, or have a poverty rate of 25 percent or more. This community-level measure is used to assess if a participant is living in a low-income community. | People live in a low-income community likely have different access to healthcare, healthcare utilization, and social needs or concerns, compared to those live in a moderate- or high-income community. |
| **Tax payer, Employed at least part-time** | A person (or business owner) that is obligated to pay taxes to a federal, state, or local government. Individual taxpayers are usually required to file and pay both federal (income tax/tax that is withheld from payroll) and state tax (taxes such as sales taxes and property tax returns) annually. Our focus is on people who have filed and paid income tax in the past XX years. Excludes people who only pay sales and property taxes. | The idea behind including taxpayers is that our tax system reflects our society’s deeper values and serves the larger common good. Taxpayers engage in this process and participation requires recognition that taxpaying is a civic duty. |
| **Civic involvement (e.g., votes in elections, not faith-based social clubs)** | People who are formal volunteers of local government, clubs, and organizations that serve to benefit the community as a whole and may have had experiences making decisions for the common good. Examples: Service organizations – adult sororities and fraternities, Lions Club, Chamber ambassadors, county boards, city commissioners, collaborative members (Family Connection, school advisory boards, PTO), youth sports coaches, etc. | People who have civic involvement have developed an understanding of community challenges and mind-sets that contribute to/address those challenges. They have had experience stepping out of “their own shoes” and seeing from another person’s/group’s perspective.  Example- Volunteer organization partners with local disabilities program to provide ramps for wheelchairs. Through this involvement, the volunteers interact with families facing disabilities and learn more about what is faced every day while navigating the community- going shopping, getting to and from school, having work options past education, etc.  Example- YMCA coach that comes from a wealthy background coaching kids from poor background and learns how transportation, work schedules of parents, lack of support for homework, etc. impact the player’s opportunities for success. |
| **Employment Status** | Community stakeholders- people with a vested professional or civic interest in impacting policy decisions so that the needs/perspectives of the people they serve are represented- such as employers, formal and informal advocates for various groups (aging, disabled, youth, etc.), land/property owners, chamber of commerce, coalition leaders, etc. | Rationale- Stakeholders would have a vested/economic interest in and experience in influencing the health and quality of life decisions that impact the community from the perspective of a specific community sector/s. |
| **Men** | Individuals who self-identify as male | By virtue of their gender, men have a physical and emotional distance from hereditary breast and ovarian cancer. They all have women in their lives who they care for but, except in rare cases, they do not personally face the threat of experiencing breast cancer. However, men can benefit from HBOC screening as there are diseases like prostate cancer and GI cancers that can affect them. |
| **Retired** | People who main sources of incomes not earned by working; individuals who have enough combined savings, investment income, and/or pension income to cover your living expenses. Typically, these people will be at a "standard" retirement age between 50-70 years old. Excludes individuals who identify as “financially independent”. | Retired individuals experience a sustained commitment to goals that are meaningful to the self and that also contribute in some way to the community, to something larger than or beyond self. Given fewer work-related commitments, retired people can engage in stable and generalized intentions to accomplish something that is meaningful to the self and of consequence to the world beyond the self.  Examples- volunteering, helping/caring for others, caring for nature and the environment, tutoring children, cleaning the neighborhood park, etc. |

**Appendix 3. Recruitment Screener 1**

| **Participant Demographics/Background** |
| --- |

1. What is your age? _______ years
2. How would you describe your gender?
   1. Male
   2. Female
   3. ____________
3. Do you identify as Hispanic, Latino, or from Spanish origin?
   1. Yes
   2. No
4. What is your race? (Select all that apply)
   1. Black or African American
   2. Native American or American Indian
   3. Asian or Pacific Islander
   4. White
   5. Other
5. Do you speak English?
   1. Yes
   2. No
6. What is the highest level of education you have completed? (Select one)
   1. Some high school
   2. High school graduate
   3. Trade school
   4. Some college
   5. College graduate
   6. Postgraduate work
   7. Prefer not to answer
7. What is your current employment status?
   1. Self-employed
   2. Employed
   3. Unemployed
   4. Retired
8. Are you currently working as a health care provider (e.g., nurse, doctor, physician assistant, nursing assistant)?
   1. Yes
   2. No
9. How long have you been living in southwest Georgia?
   1. Less than 1 year
   2. 1-5 years
   3. More than 5 years
10. What is your zip code? _______

| **Cancer history** |
| --- |

1. Have you ever been told by a health care provider that you have breast cancer?
   1. Yes
   2. No
   3. Unsure
2. If yes, in what year were you diagnosed? __________________
3. Do you currently receive medical care at a Federally Qualified Healthcare Center?
   1. Yes
   2. No
   3. Don’t know
4. Are you a member of a church?
   1. Yes
   2. No
5. If yes, in the past month, how often have you attended a church service, in person or virtually?
   1. Never
   2. Once
   3. More than once

| **Use of online services** |
| --- |

1. Do you have Internet access at home?
   1. Yes
   2. No
2. What technology devices do you have access to at home? (Select all that apply)
   1. A smart phone
   2. A desktop computer or a laptop
   3. A tablet
   4. None of the above
3. How regularly do you use email?
   1. Never
   2. Daily
   3. Weekly
   4. Monthly
4. How regularly do you take part in video conferencing on a computer (e.g., Facetime, Skype, Zoom)?
   1. Never
   2. Daily
   3. Weekly
   4. Monthly
5. How many social media sites do you have accounts with (e.g., Facebook, Twitter, online discussion forums)?
   1. 0
   2. 1-2
   3. 3-5
   4. More than 5
6. How often do you post on social media (e.g., Facebook, Twitter, online discussion forums)?
   1. Never
   2. Weekly
   3. Daily
   4. Monthly
7. How did you find out about this study?
   1. Community group: please specify
   2. Online (e.g., social media, email, etc.)
   3. Family or friend
   4. Other: please specify _______________

| **Participation in a citizen discussion group** |
| --- |

1. How interested would you be in learning about a citizen discussion group for African American citizens living in Southwest Georgia to consider whether Georgia’s health system should be making more effort to identify African Americans at highest risk for breast cancer? The conference would be held via Zoom.
   1. Not at all
   2. Somewhat/Not sure
   3. Definitely

FOR SOMEWHAT OR DEFINITELY INTERESTED: Please provide a telephone number and an email address so someone can follow-up to tell you more about the conference.

- First name
- Phone number
- Email address

1. Would you being willing to be re-contacted for research on breast cancer screening in Georgia in the future?
2. Not willing
3. Not sure
4. Willing

**Appendix 4. Recruitment Script for Screener 2**

Hello,

My name is ______________ and I am calling you regarding the Southwest GA Community Council on Hereditary Breast and Ovarian Cancer research study conducted by XXX.

[If APPROPRIATE]

_________________ at your workplace/church/social group suggested I contact you to let you know about our study.

Is this a good time to talk?

*OR*

Hello,

My name is ______________ and I am calling you regarding the Southwest GA Community Council on Hereditary Breast and Ovarian Cancer research study conducted by XXX.

Thank you for completing the survey online. I am following up with you because you indicated that you were interested in learning more about the Citizen Discussion Group we are planning. The objective for the discussion group is to engage African American citizens of Southwest Georgia in discussions about whether the state of Georgia should be making a bigger effort to offer screening for hereditary breast and ovarian cancer to African Americans.

Is this a good time to talk?

[If **NO**]

What is the best time to reach you? Is this the best number for me to use to reach you?

[STOP AND RECORD CALL BACK TIME/NUMBER ON RECRUITMENT SHEET]

[If **YES**]

Great! Let me tell you a little bit about the discussion group. The conference is part of a study being conducted with XXX collaborating with XXX.

The study is called the Southwest GA Community Council on Hereditary Breast and Ovarian Cancer.

Before I get into the specifics of the conference, I’d like to get a bit more information about you. We have up to 30 slots available for the conference. The questions I am going to ask you are aimed to make sure that the 30 participants represent different types of individuals living in Southwest Georgia.

Is it alright if I ask you a few questions to see if you are eligible for the conference?

[IF **NO**]

Thank you for taking time to talk with me today. May I ask why you have chosen not to continue our discussion of the study?

[IF **YES**]

Are you …

- - an African American man or woman
  - 25 years of age or older
  - speak English

[IF **NO**]

Thank you for taking time to talk with me today. Unfortunately, you are not eligible for the study.

[If **YES**]

My first question is:

1) Have you served as a leader of any committees for nonprofit organizations, a church, a social club, or school?

[RESPONSE OPTIONS]

[*YES / NO*]

2) How frequently do you vote in local and national elections?

[RESPONSE OPTIONS]

[*NEVER / SOMETIMES / ALWAYS*]

3) Have you ever served on a jury?

[RESPONSE OPTIONS]

[*YES / NO*]

4) Have you ever had an experience where you advocated for a particular group of people in the community?

[RESPONSE OPTIONS]

[*YES / NO*]

[RECORD ANSWERS AND ENTER IN RECRUITMENT DIVERSITY RUBRIC TABLE]

[If **NOT QUALIFIED**]

I am sorry but we have only 25 available slots. We are looking for individuals with specific sets of experiences they can bring to the discussion. May I have permission to recontact you if a slot becomes available for you?

Thank you for taking time to talk with me today.

[STOP AND RECORD *NOT QUALIFIED* IN RECRUITMENT SHEET]

[**UNSURE IF QUALIFIED**]

Thank you for answering my questions. We will review your responses as a team to determine your eligibility to participate. May I have permission to recontact after I meet with the team?

Thank you for taking time to talk with me today and I will be in touch.

[STOP AND RECORD *UNSURE QUALIFIED* IN RECRUITMENT SHEET]

[If **QUALIFIED**]

Thank you for these answers. You are eligible.

Let me tell you more about the conference to see if you continue to be interested.

The Citizen Discussion Group will be held via Zoom. The conference will begin with a 75-minute orientation on Friday March 12, and two half-day sessions on March 13 and March 20.

If you are eligible and you choose to participate you will be asked to discuss the issue of whether Georgia should be doing more to make sure that African Americans get screened for hereditary breast and ovarian cancer with other citizens from the Southwest Georgia area.

You will be presented with information from experts in the field and then have a series of discussions with other participants. At the end of the conference you will be asked to come to vote on a recommendation about African Americans being screened for hereditary breast and ovarian cancer.

In addition to the Citizen Discussion Group, you will be asked to complete 3 short surveys at different time points during the process.

The sessions will be audio recorded to make sure that your opinions and comments are documented. Participant responses will remain confidential; names and identifying information will be removed from all transcripts of the recordings.

You will be compensated a total of $200 for your time.

Do you have any additional questions?

Is this something that you are willing and able to participate in?

[IF **NO**]

Okay. I understand. May I ask why you are not will or able to participate?

Thank you for your time and consideration.

[RECORD ANSWER AND ENTER IN RECRUITMENT SHEET]

[If **YES**]

Great, what I’d like to do is to send you a fact sheet and consent form via email, and have you confirm that you plan to attend the online discussion conference. We will also ask you to respond to that email with your consent to participate.

Can I take a minute to confirm your contact information?

[RECORD ADDRESS AND ENTER IN RECRUITMENT SHEET]

Do you have any additional questions at this time that I can try to answer?

If you think of anything in the next few weeks, you can reach me at

[INSERT CONTACT INFORMATION], or you can contact our study team at

[INSERT CONTACT INFORMATION].

We look forward to seeing you at the conference this spring!

OR

I look forward to speaking with you again soon!

**Recontact Script for Participation in a Citizen Discussion Group**

Hello,

My name is ______________ and I am calling you regarding the Southwest GA Community Council on Hereditary Breast and Ovarian Cancer research study conducted by XXX.

Is this a good time to talk?

[IF **NO**]

What is the best time to reach you? Is this the best number for me to use to reach you?

[STOP AND RECORD CALL BACK TIME/NUMBER ON RECRUITMENT SHEET]

[If **YES**]

Great. I really appreciate all the time you have put into talking to me about our study recruitment and your potential participation.

You previously indicated that you wanted to be participate in the citizen discussion group.

The conference will begin with a 75-minute orientation on Friday March 12, and two half-day sessions on March 13 and March 20.

As you may recall, participants will be compensated a total of $200 for their time.

As we discussed the last time we spoke, the *Citizen Discussion Group* will include:

- Some background information on the topic and discussions with other Southwest Georgia community members about the increasing the state’s efforts to screen African Americans for hereditary breast and ovarian cancer
- There will 3-4 small group discussions (5-6 people per group) where people will share their views about the positive and negative aspects of the information presented
- At the end of the conference you will be asked to come to vote on a recommendation about the topic
- The sessions will be audio recorded to make sure that your opinions and comments are documented. Participant responses will remain confidential; names and identifying information will be removed from all transcripts of the recordings.

Are you available and still interested in the conference?

[IF **NO**]

I’m sorry to hear that. Thank you for your time.

[STOP AND RECORD ON RECRUITMENT SHEET]

[If **YES**]

Thank you.

Just to remind you, participation in this study is totally voluntary and you can change your mind at any time. I will send you a fact sheet and consent form to look at via email. We will also ask you to respond to that email with your consent to participate.

Do you have any additional questions at this time that I can try to answer?

If you think of anything in the next few weeks, you can reach me at

[INSERT CONTACT INFORMATION], or you can contact our study team at

[INSERT CONTACT INFORMATION].

We look forward to seeing you at the conference this spring!

**Appendix 5. Baseline Survey**

**1) Have you ever heard of Georgia’s hereditary breast and ovarian cancer screening program?**

- Not before today
- Yes

**2) How do you think your awareness of family history screening for breast cancer programs compares with other people in your community?**

- Much lower than others
- Lower than others
- Equal to others
- Higher than others
- Much higher than others

**3) Based on what you know now, how favorable would you be of targeting African Americans for family history screening for breast cancer?**

- Not at all
- Somewhat
- Extremely

**4) Based on what you know now, how favorable do you think people in your community would be of targeting African Americans for family history screening for breast cancer?**

- Not at all
- Somewhat
- Extremely

**5) For each of the following, please rate your level of disagreement or agreement with the statement.**

| **Broadening screening to identify African Americans at greatest risk of developing breast cancer…** | Strongly disagree | Somewhat disagree | Neither agree nor disagree | Somewhat agree | Strongly agree |
| --- | --- | --- | --- | --- | --- |
| a) would benefit me and my family. |  |  |  |  |  |
| b) would benefit my community. |  |  |  |  |  |
| c) would reduce health inequities for African Americans. |  |  |  |  |  |
| d) would have more risks than benefits. |  |  |  |  |  |
| e) is less important than other health issues facing African Americans. |  |  |  |  |  |

**6) For each of the following statements, please rate your level of willingness with the statement. *Reminder: “Hereditary” means that breast cancer may run in your family.***

| **How willing would you be to:** | Definitely unwilling | Somewhat unwilling | Neither willing or unwilling | Somewhat willing | Definitely willing |
| --- | --- | --- | --- | --- | --- |
| a) Complete a 6-question assessment regarding your family history for hereditary breast cancer? |  |  |  |  |  |
| b) Meet with a genetic counselor if you were told by a health professional that your family history shows you may be at risk for hereditary breast cancer? |  |  |  |  |  |
| c) Undergo genetic testing if a genetic counselor advised you to have your blood tested for altered genes associated with hereditary breast cancer? |  |  |  |  |  |

**7) For each of the following, please tell us the extent to which you disagree or agree with each of the statements. *Reminder: “Hereditary” means that breast cancer may run in your family.***

|  | Strongly disagree | | Somewhat disagree | Neither agree nor disagree | Somewhat agree | Strongly agree |
| --- | --- | --- | --- | --- | --- | --- |
| a) My opinions can influence how the state of Georgia screens for hereditary breast cancer. |  |  | |  |  |  |
| b) Georgia’s health policy makers want to hear my opinion about how screening for hereditary breast cancer should be conducted. |  |  | |  |  |  |
| c) Only Georgia’s health policy makers can decide how screening for hereditary breast cancer should be conducted. |  |  | |  |  |  |
| d) Community members working together can influence Georgia’s health policy makers’ views on screening for hereditary breast cancer. |  |  | |  |  |  |
| e) My opinions are not important to Georgia’s health policy makers who decide about screening for hereditary breast cancer. |  |  | |  |  |  |
| f) I want my voice to matter in Georgia's health policy makers’ decisions about screening for hereditary breast cancer. |  |  | |  |  |  |

**8) For each of the following, please rate your level of disagreement or agreement with the statement.**

| **I can explain to members of my community...** | Strongly disagree | Somewhat disagree | Neither agree nor disagree | Somewhat agree | Strongly agree |
| --- | --- | --- | --- | --- | --- |
| a) how a person’s ancestry can influence their risk for breast cancer. |  |  |  |  |  |
| b) how a person’s family history can influence their risk for breast cancer. |  |  |  |  |  |
| c) how altered genes might influence risk for breast cancer. |  |  |  |  |  |
| d) why some people should be given priority for breast cancer screening because of their ancestry. |  |  |  |  |  |
| e) the difference between family history and ancestry. |  |  |  |  |  |

**9) The next section is about your general experiences with the health care system. The health care system includes medical or hospital visits, health insurers, and medical research.**

**For each of the following, please rate the extent to which you disagree or agree with the statement.**

|  | Strongly disagree | Somewhat disagree | Neither agree nor disagree | Somewhat agree | Strongly agree |
| --- | --- | --- | --- | --- | --- |
| a) The health care system has put my medical needs above all other considerations when treating my medical problems. |  |  |  |  |  |
| b) In my experience, I have received high-quality service from the health care system. |  |  |  |  |  |
| c) The health care system cares more about holding costs down than it does about meeting my health needs. |  |  |  |  |  |

**10) For each of the following, please rate your ability to communicate in each of the situations described below on a scale from not at all able to fully able**

| **To what extent are you able to:** | Not at all able | Somewhat able | Fully able |
| --- | --- | --- | --- |
| a) Share your opinions about a topic with a small group of strangers. |  |  |  |
| b) Summarize what someone has shared in a conversation. |  |  |  |
| c) Report the results of a small group discussion to a large group of strangers. |  |  |  |
| d) Express your disagreement with others opinions in a small group discussion. |  |  |  |
| e) Have a discussion about a topic with people who have different viewpoints than yours. |  |  |  |
| f) Share your opinions about a topic with a large group of strangers. |  |  |  |

**Appendix 6. Post-Deliberation Survey**

**1) How do you think your awareness of family history screening for breast cancer programs compares with other people in your community?**

- Much lower than others
- Lower than others
- Equal to others
- Higher than others
- Much higher than others

**2) Based on what you know now, how favorable would you be of targeting African Americans for family history screening for breast cancer?**

- Not at all
- Somewhat
- Extremely

**3) Based on what you know now, how favorable do you think people in your community would be of targeting African Americans for family history screening for breast cancer?**

- Not at all
- Somewhat
- Extremely

**4) For each of the following, please rate your level of disagreement or agreement with the statement.**

| **Broadening screening to identify African Americans at greatest risk of developing breast cancer…** | Strongly disagree | Somewhat disagree | Neither agree nor disagree | Somewhat agree | Strongly agree |
| --- | --- | --- | --- | --- | --- |
| a) would benefit me and my family. |  |  |  |  |  |
| b) would benefit my community. |  |  |  |  |  |
| c) would reduce health inequities for African Americans. |  |  |  |  |  |
| d) would have more risks than benefits. |  |  |  |  |  |
| e) is less important than other health issues facing African Americans. |  |  |  |  |  |

**5) For each of the following statements, please rate your level of willingness with the statement. *Reminder: “Hereditary” means that breast cancer may run in your family.***

| **How willing would you be to:** | Definitely unwilling | Somewhat unwilling | Neither willing or unwilling | Somewhat willing | Definitely willing |
| --- | --- | --- | --- | --- | --- |
| a) Complete a 6-question assessment regarding your family history for hereditary breast cancer? |  |  |  |  |  |
| b) Meet with a genetic counselor if you were told by a health professional that your family history shows you may be at risk for hereditary breast cancer? |  |  |  |  |  |
| c) Undergo genetic testing if a genetic counselor advised you to have your blood tested for altered genes associated with hereditary breast cancer? |  |  |  |  |  |

**6) For each of the following, please tell us the extent to which you disagree or agree with each of the statements. *Reminder: “Hereditary” means that breast cancer may run in your family.***

|  | Strongly disagree | Somewhat disagree | Neither agree nor disagree | Somewhat agree | Strongly agree |
| --- | --- | --- | --- | --- | --- |
| a) My opinions can influence how the state of Georgia screens for hereditary breast cancer. |  |  |  |  |  |
| b) Georgia’s health policy makers want to hear my opinion about how screening for hereditary breast cancer should be conducted. |  |  |  |  |  |
| c) Only Georgia’s health policy makers can decide how screening for hereditary breast cancer should be conducted. |  |  |  |  |  |
| d) Community members working together can influence Georgia’s health policy makers’ views on screening for hereditary breast cancer. |  |  |  |  |  |
| e) My opinions are not important to Georgia’s health policy makers who decide about screening for hereditary breast cancer. |  |  |  |  |  |
| f) I want my voice to matter in Georgia's health policy makers’ decisions about screening for hereditary breast cancer. |  |  |  |  |  |

**7) For each of the following, please rate your level of disagreement or agreement with the statement.**

| **I can explain to members of my community...** | Strongly disagree | Somewhat disagree | Neither agree nor disagree | Somewhat agree | Strongly agree |
| --- | --- | --- | --- | --- | --- |
| a) how a person’s ancestry can influence their risk for breast cancer. |  |  |  |  |  |
| b) how a person’s family history can influence their risk for breast cancer. |  |  |  |  |  |
| c) how altered genes might influence risk for breast cancer. |  |  |  |  |  |
| d) why some people should be given priority for breast cancer screening because of their ancestry. |  |  |  |  |  |
| e) the difference between family history and ancestry. |  |  |  |  |  |

**8) The next section is about your general experiences with the health care system. The health care system includes medical or hospital visits, health insurers, and medical research.**

**For each of the following, please rate the extent to which you disagree or agree with the statement.**

|  | Strongly disagree | Somewhat disagree | Neither agree nor disagree | Somewhat agree | Strongly agree |
| --- | --- | --- | --- | --- | --- |
| a) The health care system has put my medical needs above all other considerations when treating my medical problems. |  |  |  |  |  |
| b) In my experience, I have received high-quality service from the health care system. |  |  |  |  |  |
| c) The health care system cares more about holding costs down than it does about meeting my health needs. |  |  |  |  |  |

**9) For each of the following, please rate your ability to communicate in each of the situations described below on a scale from not at all able to fully able.**

| **To what extent are you able to:** | Not at all able | Somewhat able | Fully able |
| --- | --- | --- | --- |
| a) Share your opinions about a topic with a small group of strangers. |  |  |  |
| b) Summarize what someone has shared in a conversation. |  |  |  |
| c) Report the results of a small group discussion to a large group of strangers. |  |  |  |
| d) Express your disagreement with others opinions in a small group discussion. |  |  |  |
| e) Have a discussion about a topic with people who have different viewpoints than yours. |  |  |  |
| f) Share your opinions about a topic with a large group of strangers. |  |  |  |

**On a scale of 1 to 10, please select the number to indicate your answer:**

|  | 1 (Not at all) | 2 | 3 | 4 | 5 | 6 | 7 | 8 | 9 | 10 (Very much) |
| --- | --- | --- | --- | --- | --- | --- | --- | --- | --- | --- |
| 10) Do you feel that your opinions were respected by your group? |  |  |  |  |  |  |  |  |  |  |
| 11) Do you feel you were listened to by your facilitator? |  |  |  |  |  |  |  |  |  |  |
| 12) Do you feel that the process that led to your group’s responses was fair? |  |  |  |  |  |  |  |  |  |  |
| 13) How willing are you to abide by the group’s final position, even if you personally have a different view? |  |  |  |  |  |  |  |  |  |  |

**In this next section, please tell us how this experience went for you.  Responses will be used to improve future research studies.**

**14) For each of the following, please select the number to indicate your answer on a scale of 1 (not at all) to 10 (very much).
 
How much did attending the discussion sessions change your...**

|  | 1 (Not at all) | 2 | 3 | 4 | 5 | 6 | 7 | 8 | 9 | 10 (Very much) |
| --- | --- | --- | --- | --- | --- | --- | --- | --- | --- | --- |
| a) understanding about Georgia’s family history screening for breast cancer program? |  |  |  |  |  |  |  |  |  |  |
| b) opinion about Georgia’s family history screening for breast cancer program? |  |  |  |  |  |  |  |  |  |  |

**15) On a scale of 1 to 10, please rate how helpful you found the following:**

|  | 1 (Not helpful at all) | 2 | 3 | 4 | 5 | 6 | 7 | 8 | 9 | 10 (Extremely helpful) |
| --- | --- | --- | --- | --- | --- | --- | --- | --- | --- | --- |
| a) Question and answer interaction with team members. |  |  |  |  |  |  |  |  |  |  |
| b) The expert video presentations. |  |  |  |  |  |  |  |  |  |  |
| c) Discussing the issues with other participants. |  |  |  |  |  |  |  |  |  |  |

**16) How much would you expect to be compensated for participating in a similar conference in the future?**

________________________________________________________________

**17) In the future, if the conference is held in-person instead of online, how willing would you be to participate?**

- Very unwilling
- Unwilling
- Not sure
- Willing
- Very willing

**18) How willing would you be to participate in a similar conference on another topic?**

- Very unwilling
- Unwilling
- Not sure
- Willing
- Very willing

**19) Please provide any other suggestions for improving the conference:**

________________________________________________________________

**Appendix 7. Qualitative Evaluation of Small Group Deliberations**

| **Codes** | **Definition** | **Example Quotes** |
| --- | --- | --- |
| **Understanding of analytic information** | | |
| Confirmation, clarification, checks for accuracy: content | Speaker re-states or reflects back information that he or she has heard from another participant for the purpose of checking for accuracy of information, or for confirming a shared understanding of the facts or issues being discussed.    Apply this code if the statement is related to deliberation content. | “I wanted to ask a question, that only one percent of all those people that they originally screened out of that – I think 24,000. Only one percent of those people – was that “had the BRCA gene?” Is that what it said?”    “Maybe I missed something but that’s what it looked like to me that it showed that beyond a shadow of a doubt that we are more at risk, the African American descent. Is that right?”    “Can I maybe just resay it, just to make sure that I understood what you were saying. But the awareness of using this tool that’s based on past history, that by looking at past history, you can learn a lot about if you’re at an increased risk” |
| Confirmation, clarification, checks for accuracy: procedure | Apply this code if the statement is related to deliberation procedures, or notes editing process. | “We just vote one time, right? For the top one and top two?”    “Where it says, “Screening needs to move out of the public health sector,” that's not quite it. It needs to stay in the public health sector. It just needs to be added to more than the public health sector.” |
| Agreement with peers | Participant shows agreement/disagreement with their peers or refers to statements made by their peers. | “XX said it, but I understood and agreed with her on that aspect because that is how we think.”    “That’s exactly what I was saying, Ms. XX.”    “I agree with what he stated about the insurance companies using that information to either deny insurance or give inflated prices.” |
| **Reasoned justification of ideas** | | |
| Applying the information presented: general | Participant refers to expert testimonies to explain a pro or con they are offering. Statements needs to be related to testimonies. We do not judge the accuracy of the information. | “We mentioned in the film that sometimes, the insurance pays for it, and sometimes it doesn't. One of the negative things about anytime you involve insurance, to me, is that you also have the possibility of them dropping you or significantly raising your premiums. It's almost as if you would avoid going to be tested because you don't wanna risk the possibility of either one of those occurring.”    “We’re still not talking about the males. If they’re presenting information about males in there, why are we not talking about them as well. And I know it’s about females having breast cancer but there is still that small percentage of males that get breast cancer.” |
| Applying the information presented: genetic information | Apply this code if the statement is about genetics and inheritance, and the difference between family history and ancestry. | “That’s how I was looking at that on that last question that she had posed. But some of my pros was this. The pros was the awareness in itself. I liked the fact that making it aware because a lot of people will not get the screening if they don’t think that it pertained to them specifically. And how we identify the risk factors and looking at the hereditary trait that may be me, will target me. So, that’s gonna prompt my interest and wanna get it done more quickly and readily. And using that same scenario with a vehicle. If I know that, wait a minute, I’ve got a history of my brakes going out. I need to get in there and get it changed even though I haven’t had a problem.”    “If it’s a false positive, it could cause people to worry unnecessarily. Also, it could cause you to be out getting all kinds of testing and things done that’s unnecessary. If it’s a false negative, you could lose the benefit of treatment early on because you think you’re okay. I was saying the uncertainty of the screening results was definitely a top con.” |
| Considering both sides of an issue | Participant offers a counterpoint or counterview to a pro or con to make sure all views are considered | “A false negative, you will lose the insight and not get anything done because you think you’re fine. Then, if you’re a false positive, you think you got it, and you’re doing all these unnecessary things, could possibly be getting discriminated on your insurance.”    “Part of that is going to be a pro. Part of that is going to be a con. The positive part is that now we know they need treatment. The next step will be, “Now, how do we find that treatment? How do we get them into the treatment? Can they afford it? Is it even available in that community?” All that ripples after that.” |
| **Adoption of a societal perspective** | | |
| Societal perspective | Participant raises a pro/con based on group-level benefit or harm.  Participant specifically states that an approach might not be appealing or useful to the individual participant but could be beneficial to the larger community. | “All women need to have equal access to this knowledge so that, if you’re gonna really impact something as serious as cancer for women, then you can't be selective. It can't be isolated into just the public health arena.”    “And the reason I said yes was because the research study could, actually, identify some commonalities among African Americans. And it could also identify some early onset of some reasons that breast cancer may appear in African Americans early. So, the research study would be good for that. I also said that it could, actually, add to the awareness of research since there were not a lot of research studies done for African Americans maybe in the past 10 or 15 years. So, now doing research studies for African Americans is definitely a benefit.”    “Well, the trust of the government system in itself, I think would be. You've got to be willing to trust, and with our history with our government and with people testing us and screening us as African Americans, it's hard not to remember those kinds of things and to be openly trusting.” |
| Economic costs | Participant considers issue from the perspective of cost to a group | “The insurance would come into play at some point where they may want to charge higher premiums for someone who does have that hereditary factor in.”    “I don’t know the exact financial cost of this program, but it could affect others by the cost of it. Insurance may not be able to cover it. This could reduce others from getting screened or even doing something about it.” |
| Personal interests | Statements indicating personal interests to justify or make a point about something/policy/opinion | “Another thing would be populations with these mutations would be able to make more informed healthcare decisions. I know to increase my health insurance, I would know to make more informed decisions about my diet. “    “It’s not just getting people to use the service. They don’t know it’s there. Because like I say, if it wasn’t for the fact that I worked in the child protective agency field and worked with the health department, I wouldn’t have known that that service was there for helping people who have exposure to breast cancer, ovarian cancer. I wouldn’t have known any of that.” |
| Community agency | Statements that reflect we-ness linked to action. | “And even in our community, in the community of Albany, Georgia, I think that all genders and races can benefit from it because this is a low-income area here that we’re living in. So, to a certain extent, the community is underserved as well.”    “And that’s why we have targeted to get even more information that we can because I think the more information that we have, the more we’d be advised on what direction we need to go and how and how early. We may even need to start screening earlier than 50.” |
